# Comparison of Four Yogic Breathing Techniques Regarding Their Impact on Heart-Rate Variability and Breathing Frequency

**DOI:** 10.3390/medsci14060604

**Published:** 2026-09-25

**Authors:** Janika Epe, Rudolf Stark, Bertram Walter, Ulrich Ott

**Affiliations:** 1 Bender Institute of Neuroimaging, Department of Psychotherapy and Systems Neuroscience, Justus Liebig University Giessen, Otto-Behaghel-Str. 10H, 35394 Giessen, Germanyott@igpp.de (U.O.); 2 Institute for Frontier Areas of Psychology and Mental Health, Wilhelmstr. 3A, 79098 Freiburg im Breisgau, Germany

**Keywords:** breathing, HRV, Ujjayi, Paced Breathing, Kapalabhati, Alternate Nostril Breathing

## Abstract

Background: The manner of breathing affects the autonomic nervous system (ANS) and corresponding processes in the body. Most studies focus on techniques prolonging breathing. Nevertheless, there are also techniques aimed at accelerating or changing other aspects of the breath. The variety of concepts suggests that different breathing techniques could have different effects on the ANS. This study intended to investigate differential effects of four yogic breathing techniques on heart-rate variability: (1) Ujjayi—relaxation through slowing down the breath; (2) Paced Breathing—enhancement of concentration by following a precise protocol of slowdown breathing; (3) Kapalabhati—raising wakefulness by mild hyperventilation; and (4) Alternate Nostril Breathing—balancing the autonomic nervous system by alternating breath between the nostrils. We hypothesized that there would be (1) differences in the impact on baseline measures after the training weeks, (2) differences between baseline and practice, (3) higher impact with longer performance of the breathing technique, and (4) differences between techniques. Methods: This study was conducted with 36 participants who learned each technique in group sessions and practiced them daily for two weeks of an eight-week program. After practicing each technique, participants performed the breathing technique while HRV, breathing frequency and other psychological variables were recorded; rMSSD was calculated for to interpret the HRV. Results: We found significant differences between calming and accelerating breathing techniques on HRV and breathing frequency, and different effects of the four breathing techniques compared to their baseline and over practice time. Conclusions: Because breathing is such an easily accessible tool for regulation, it seems important to see which breathing technique helps people regulate quickly and effectively in everyday life.

## 1. Introduction

The enhancement of an individual’s capacity for self-regulation when dealing with stressful situations is of great importance. Many studies address this by investigating the effects of contemplative practices, such as Yoga practice, meditation, and other consciousness-based practices, as well as biofeedback and neurofeedback. A common mechanism in most stress-regulating interventions is the modulation of physiological processes via changes in breathing frequency [1,2]. Breathing is a key mechanisms for actively regulating the body’s stress-responses because it influences the autonomic nervous system (ANS) and heart rate (HR) [3]. While breathing in, there is a dominance of the sympathetic nervous system (SNS) through an inhibition of the vagal outflow. This dominance leads to a higher HR [4], a higher muscle tone, and higher blood pressure. During exhalation, the dominance of the SNS lowers, so that the influence of the parasympathetic nervous system (PNS) increases, leading to a lower HR, and reduction of muscle tone and blood pressure [5]. This oscillation of HR, called respiratory sinus arrhythmia, is one component of heart-rate variability (HRV). The SNS is associated with the fight-or-flight response, while the PNS is associated with recovery and relaxation [4]. Breathing parameters also influence the autonomic response to threat, whereas a slower breathing frequency leads to a reduced response to stress [6].

Different parameters in breathing could be actively modified by everyone to explore the influence of breathing on autonomic processes, for example, extending or shortening inhalation or exhalation separately; extending or shortening the pause between inhalation and exhalation; focusing on a regular breath flow with similar inhalation and exhalation; or reducing or accelerating breathing rhythm in general. Techniques using a slower breathing rhythm typically lead to a relaxation effect, whereas techniques that accelerate breathing frequency lead to an activating effect [7]. Breathing techniques are found in many different traditions of exercises designed to improve health and well-being. In this article, we focus on the many ways to observe and control the breath in yogic traditions. On the one hand, these techniques are used to support physical exercises; on the other hand, they are practiced as standalone exercises to influence the body and mind. Even though studies postulate that underlying neurophysiological mechanisms influence the practicing person regardless of the technique used [8,9], many studies focus on the specific effects of breathing techniques.

One yogic breathing technique is called Ujjayi. It helps prolong the breathing phases by constricting the vocal cords to increase airway resistance. This technique helps to practice controlled, deep breathing, which is known to have a great impact on emotion regulation and dealing with negative psychological states [10]. It also aids in the regulation of autonomic functions [11] and dealing with motion sickness [12]. Pal et al. (2004) [13] found that short-term practice of slow breathing also increases parasympathetic activity and decreases sympathetic activity. They measured a significant reduction in basal heart rate and therefore assumed an enhancement of vagal activity [13].

Another method to downregulate breathing rhythm is called Paced Breathing. Within this technique, all four phases of the breathing cycle are guided evenly or are systematically extended: (1) inhalation, (2) pause after inhalation, (3) exhalation, and (4) pause after exhalation. The lengthening of the breath can be supported by the use of audio files [14]. One advantage of direct guidance with audio files or a metronome is that one can actively breathe in the so-called resonance frequency. Resonance breathing is breathing at a frequency of about 10 s per breath, consisting of 4 s breathing in and 6 s breathing out. It is called resonance breathing due to the resonance between breathing frequency and Mayer waves (in HRV 0.1 Hz), which play a role in blood pressure regulation. It has been discovered that this is a breathing rhythm at which baroreflex sensitivity and HRV increase strongly, considered indicative of good autonomic regulation [15].

While slowing down breathing rhythm has a relaxing, balancing effect on the ANS, accelerating the breathing frequency might lead to the opposite effect, sometimes seen when people hyperventilate. A faster breathing rhythm activates the central nervous system (CNS). Techniques to accelerate breathing are also known as a “cleaning” technique (kriya) in yogic traditions, called Kapalabhati and bhastrika. They use active movement of the abdomen during exhalation (Kapalabhati) or during exhalation and inhalation (bhastrika) to accelerate airflow out of and into the lungs. Lalitha et al. (2020) [16] found a significant parasympathetic reduction when analyzing the rMSSD in HRV (Root Mean Square of Successive Differences) after a session of Kapalabhati, the technique for hyperventilation used in the present study. The effect shifted toward parasympathetic dominance after 20 min of resting time [16], speaking for a period of higher activation, followed by a relaxing effect. Other studies could not show an effect of fast breathing techniques on autonomic variables [13,17]. At the same time, enhanced alpha amplitudes during this practice were reported [18]. Sharma et al. (2014) [19] found that breathing techniques had a positive effect on executive functions. When differentiating between slow and fast breathing techniques, fast breathing techniques showed an additional, supportive effect [19].

Another way to influence the breathing process is by breathing alternating through the nostrils (nadi shodhana, anuloma viloma). When practicing, one nostril is closed manually so that the air streams through the other nostril. While unilateral nostril breathing means using only one nostril for a practice cycle, we used the alternating nostril breathing technique where the used nostril changes after every breathing cycle. Studies are showing different effects depending on the nostril used for breathing [20]: a balancing effect was found for breathing alternating through the nostrils. At the same time, there was a sedative effect for breathing through the left nostril and an activating effect for breathing only through the right nostril. This was shown through higher sympathetic arousal during right unilateral nostril breathing and parasympathetic dominance in left unilateral nostril breathing after six weeks of training, compared to normal breathing [18]. The balancing effect was also shown in EEG studies, where Alternate Nostril Breathing (ANB) affected lateralization between the left and right hemispheres [21]. Other effects showed enhanced parasympathetic regulation, leading to reduced blood pressure and heart rate.

When looking at the above-mentioned results of breath regulation, the question arises whether breathing techniques could be individually used to regulate a person’s arousal state in different types of situations, like a toolbox for self-regulation [22]. When looking at psychological effects, we found, indeed, individual profiles for the breathing techniques after training. Ujjayi, for example, showed a relaxing effect, whereas Paced Breathing resulted in a higher awareness of inner experiences (as described by the participants). In contrast, Kapalabhati led to more vitality and joy of life [22]. Other studies comparing physiological effects found slight differences between techniques that prolong the breathing phases, like square breathing, 4-7-8-breathing, and breathing at a rate of 6 beats per minute (bpm), with the 6 bpm breathing increasing HRV the most [23] and no changes in mood variables for any techniques. Reviews also showed that slow breathing has a beneficial effect on HRV parameters, indicating enhanced cardiac autonomic regulation (i.e., [24]).

In our previous study [22], we examined one fast and three slow breathing techniques and their effect on subjective experience in one group of participants. We found slight differences between the techniques. In this article, we now focus on effects on physiological variables: HRV and breathing frequency. The techniques studied were (1) extension of exhalation with Ujjayi (resistance breathing); (2) audio-guided systematic extension of inhalation, exhalation, and pauses in between (Paced Breathing); (3) high-frequency breathing driven by an active and fast exhalation, called Kapalabhati (bellows breath); and (4) breathing alternating through both nostrils (ANB).

### Hypothesis

Moreover, we wanted to examine whether an eight-week breathing training in general has a sustained effect on the HRV, independent of the practiced techniques.

We hypothesized that (1) an eight-week breathing training, in general, would have an effect on baseline HRV and breathing frequency compared to baseline measures before the training starts.

Looking at the four different breathing techniques, we were interested in their different impact on HRV, while also considering their impact on breathing frequency. We hypothesized that (2) calming breathing techniques would lead to a higher HRV during practice, compared to the baseline measurement right before the practice (spontaneous breathing during a relaxed state).

To check how quickly a significant effect can be achieved, we examined whether there is a difference between the first and second part of the intervention measurement, assuming (3) a higher HRV in the second half of the measurement. To identify differences between the effects of the four breathing techniques, we conducted an exploratory comparison of the effects of the breathing techniques on heart-rate variability and respiratory rate.

The analyses were also carried out on the respiratory-rate data in order to identify differences and similarities between the effects on respiratory rate and HRV.

## 2. Materials and Methods

The materials, setup, and participants are similar to our previous study [22]; all relevant information is provided in this section.

This study was ethically approved by the ethics committee of the Faculty of Psychology and Sport Science of the Justus Liebig University Giessen, Germany. The identification code was 2017-0002 (date of approval: 5 April 2017). This study followed German national legislation. Informed consent was obtained from all subjects involved in this study.

### 2.1. Participants

Participants were recruited using a mailing list sent to students and employees of the university. Due to practical restrictions regarding the room for conducting the training and the time required for physiological measurements (see below), the maximum number of participants had to be limited to n = 36. Thirty-six participants started the measurements (18 males, 18 females, mean age of 28.6, SD = 8.9, and range of 21–63); twenty-four were university students, and twelve were employees. Two participants completed only three baseline measurements and two measurements with a breathing practice; two women quit after the baseline measurement because of time issues. Thirty-two completed the whole training (*n* = 32 for analysis of HRV; *n* = 34 for breathing frequency). Twenty-six subjects (16 males, 10 females, mean age of 29.7, SD = 9.9, and range of 21–63) participated in a follow-up measurement after six months. The other participants did not respond to the invitation to the follow-up measurement or could no longer arrange a measurement due to relocation. Follow-up data were collected but could not be analyzed due to technical issues. Exclusion criteria were the diagnosis of epilepsy (also in first-degree relatives), lung dysfunction, acute psychotherapeutic treatment, and prior experience with yogic breathing techniques.

### 2.2. Breathing Training and Study Design

For the data collection, a multiple-baseline design was employed [25]. Participants were randomly assigned to three groups of twelve persons each, and they started the training at three different time points. The training in the different breathing techniques is referred to as “intervention” because we conceptualized them as active self-regulation techniques that participants could apply themselves in order to change their mental state as desired.

All participants took part in the first baseline measurement (week one), and twelve participants immediately started the training in week two (first intervention: Ujjayi; group 1). After two weeks of training or waiting, all participants were measured again in week four, either for their second baseline measurement (groups 2 and 3) or for an intervention measurement (group 1). Afterward, the twelve participants belonging to group 1 continued the training with one of the other three interventions (they were randomly assigned to a specific sequence of the remaining three interventions; four participants per training session), and another twelve started the training (group 2) with the first intervention (Ujjayi). After another two weeks of training (weeks five and six), all participants were measured again in week seven of the program (group 3: baseline; groups 1 and 2: intervention measurements). After that, the third group started the training, too, with the first intervention (Ujjayi); the others continued with one of the other three different interventions (members of group 2 had their training sessions together with members of group 1). Throughout the entire study, two weeks of training alternated with one week of measurements, in which all participants were measured once. Therefore, group 1 had one baseline measurement before the training started, group 2 had two baseline measurements, and group 3 had three baseline measurements before the training started (see Figure 1). For a description of the procedure during the measurements, see Section 2.3.

The pure training without measurements was an eight-week program with four modules, training different yogic breathing techniques (see Figure 1, light blue blocks). Each breathing technique was taught in two weeks, with group sessions, additional video-learning material, and worksheets. The 60 min sessions were held in a yogic classroom with four to twelve participants. The instructor had undergone training for two years to become a hatha yoga teacher and had one and a half years of teaching and ongoing supervision experience. Most postures were taught during the first intervention; only small adjustments were made during the other interventions, as more focus was given to teach new breathing techniques. The description of the breathing training was published as a book (in German) [26]. The audio files used for Paced Breathing and the video tutorials showing the asanas taught can be downloaded free of charge from a website [27].

During the first intervention (Ujjayi), participants had two group sessions per week; the other interventions included one group session and one online tutorial per week. Participants had a structured plan with a daily routine to practice until the next session. Every exercise they learned in the sessions or tutorials was available online as tutorial videos. Participants were required to practice their breathing techniques daily and to record their practice. The training always started with the same breathing technique (Ujjayi); the other three techniques followed in different sequences (fully permuted across all participants). As mentioned previously, the training sessions were 60 min long; the daily routine for their home practice took about 20 min to complete.

#### 2.2.1. Ujjayi

This intervention formed the basis of the program, in which participants learned yogic postures, felt their breath in different parts of the body, combined movement-flows with the breathing rhythm, and learned the Ujjayi-breathing technique. It included four group sessions and video-based training between the sessions.

Participants were taught how to feel their body, their movements, and their breathing, and bring them all together. They were instructed to get to know and accept their boundaries and adapt the exercises to their ability level, i.e., being aware of themselves without evaluating. With the possibility of adaptation, it was ensured that they had a regular and steady breathing rhythm, i.e., not too fast or unsteady, which may be caused by overextension.

By practicing this technique, the participants learned how to use the movement of their breath in the body to support postures and movements. One underlying principle was to differentiate between movements that reduce lung volume and movements that increase volume of the lungs (“opening” and “closing” movements). The goal was to find and perform the movements so that the breath could adapt its rhythm and flow freely. Participants practiced an average of M = 4.99 times a week, plus two training sessions.

#### 2.2.2. Paced Breathing

In this intervention, participants learned to prolong their breathing cycle based on audio files. They became aware of the length of inhalation and exhalation and the pause in between (four phases of breathing: inhalation–holding–exhalation–holding). The audio files guided participants to prolong their breath at a 1:2 or 2:3 ratio of inhalation and exhalation. The breath was guided at a ratio of 1:2 from a three-second inhale and six-second exhale to a maximum of a six-second inhale, four-second hold, twelve-second exhale, and four-second hold. At the 2:3 ratio, it was guided from a four-second inhale and six-second exhale to a maximum of a six-second inhale, four-second hold, nine-second exhale, and four-second hold.

Participants used a ratio closer to their natural breathing rhythm. Files were created with the program “Audacity” (Version 2.1.2; The Audacity Team, 2016). Similar to a former study using Paced Breathing [14], we used the same frequency of 330 Hz to indicate the inhalation phase and 262 Hz for exhalation. Participants practiced an average of M = 4.06 times a week, plus two training sessions.

#### 2.2.3. Kapalabhati

In this intervention, participants learned how to accelerate the breath and pause it. They practiced a mild and controlled form of hyperventilation, followed by a spontaneous pause in breathing. After they had learned to pay specific attention to the movement of the breath in the abdomen, they were asked to focus on the moment after exhalation. They were instructed not to inhale automatically, but to wait until their body gave them the impulse for inhalation, resulting in a longer pause between exhalation and inhalation. Afterward, they learned to intensify exhalation by tensing the abdomen at the end of the exhalation and relaxing it consciously before inhalation, so that air could flow into the lungs; this resulted in active exhalation and passive inhalation. By practicing this, they learned how to increase their breathing rhythm up to one breath per second. They took 20–60 breaths per minute, increasing the number slowly based on their level of experience. Participants practiced an average of M = 3.79 times a week, plus two training sessions.

#### 2.2.4. Alternate Nostril Breathing (ANB)

During this intervention, participants learned the “alternating nostril breathing” technique. They started by taking one breath (exhale–inhale) through both nostrils, and then closing the right nostril with the thumb of the right hand (in the pause between inhalation and exhalation) and breathing out and in through the left nostril. After breathing in, they closed the left nostril with the ring finger of the right hand and opened the right one, breathed out and in, and closed the right nostril, and then opened the left one to breathe out and breathe in again. In this way, they took 16–32 breaths, increasing the number slowly based on their level of experience. Participants practiced an average of M = 3.72 times a week, plus two training sessions.

### 2.3. Measurements

For the baseline measurements, participants sat in a chair in a dark room with a small light and closed their eyes. For 10 min, they were left alone without any instructions (resting state) while physiological recordings were made, including breathing rhythm frequency and the electrocardiogram (ECG) (see Section 2.4). In addition to the recordings presented in this study, a dry EEG cap was applied. Results are not reported here. Afterward, participants completed different questionnaires and an attention network test online.

Intervention measurements examined the effect of the intervention learned in the previous two weeks. Similar to the baseline measurements, participants sat in a chair in the same room with standardized environmental conditions. All measurements were taken at the same time of day, and participants were asked to maintain their usual routines and report any deviations (regarding caffeine intake, nicotine intake, diet, and sleep). First, they were asked to sit still with their eyes closed for 5 min to record a resting state. Afterward, they were instructed to perform the breathing technique they learned in the two weeks before for 10 min. Throughout the recording, the physiological parameters were recorded. The first and last minute of the recording were removed, and the average of the remaining eight minutes was calculated. For comparisons within one breathing technique, the eight minutes were divided into two four-minute blocks. Then, questionnaires were filled out, referring to the last two weeks.

Applied questionnaires were the Comprehensive Inventory of Mindfulness Experiences (CHIME) [28], the Perceived Stress Questionnaire (PSQ) [29], and a questionnaire on the positive aspects of current physical well-being (FAW) [30]; for further details see Epe, Ott, and Stark (2021) [22]. At the end of their last intervention measurement, participants completed a questionnaire about all interventions and the benefit of using them in everyday life.

After six months, some participants took part in a follow-up measurement. At this appointment, baseline was recorded for 5 min first, followed by 5 min of every breathing technique they learned during the training. Techniques were recorded in the same order the participants had learned them during the training. Breathing rhythm and ECG were recorded.

### 2.4. Physiological Variables

ECG and breathing rhythm were recorded with an actiChamp amplifier from BrainProducts (Gilching, Germany). ECG was recorded with three electrodes on the chest with a sampling rate of 500 Hz and a 24-bit sampling resolution with an input voltage range of 4.8 V, the same as for the breathing data.

The breathing rhythm was measured using a respiratory-belt around the abdomen; the location was selected based on the natural breathing movement. Collected raw ECG and breathing data were loaded into EEGLAB [31] within MATLAB Version 9.1 (1994–2016, Natick, Massachusetts, USA: The MathWorks, Inc). The data were extracted for further processing with Kubios HRV [32] and Matlab.

#### 2.4.1. Heart-Rate Variability (HRV)

The extracted data were preprocessed and analyzed with Kubios HRV. For artifact correction, each recording was checked manually, looking for abnormal RR intervals and missed peaks. Afterward, to standardize preprocessing and check for missed errors, the “very low” threshold-based artifact correction within Kubios was used, applying a 0.45 threshold level to the data. This preprocessing followed recommendations from Laborde, Mosley, and Thayer (2017) [33].

The rMSSD (Root Mean Square of Successive Differences) was extracted from the generated results file for further evaluation in SPSS Statistics 31. The rMSSD multiplies the time difference between RR intervals (to generate only positive values), sums them, and divides the sum by the number of RR intervals for a mean value, from which the square root will be reported. Based on Shaffer and Ginsberg (2017), standard values for the rMSSD were applied, and data outside the range between 19 ms and 75 ms were excluded [33,34]. We chose rMSSD as a time-domain HRV parameter because it is relatively free of respiratory influences [33]. It correlates strongly with HF power and pNN50, other time-domain values.

$$RMSSD=sqrt\left(\frac{1}{N-1}*\sum_{i=1}^{N-1}{\left({RR}_{i+1}-{RR}_{i}\right)}^{2}\right)$$

RR_i_: The duration of a specific interbeat interval in milliseconds.

RR_i+1_: The immediately following interbeat interval.

N: The total number of analyzed intervals.

#### 2.4.2. Breathing Rhythm

The extracted data were further processed with MATLAB. No general amplitude threshold was applied; it was adaptive to the generated signal, depending on the position of the respiratory-belt and the strength of breathing movement. Peak analysis was applied with a minimum distance of 20 ms between peaks. Afterward, peaks per minute were calculated for the analyzed time windows.

### 2.5. Data Analyses

Data were analyzed using IBM SPSS Statistics 31 for the mixed models. Mixed procedure was applied because of several missing values. Within mixed models, all values were retained, and no participants were listwise excluded.

Available data led to four values per intervention: t1 being the baseline measure before training the specific intervention, t2 being the baseline measure after training the intervention, t3 being the first part of the measurement while practicing the intervention, and t4 being the second part of the measurement while practicing the intervention (see also Figure 2).

To test the different hypotheses, linear mixed models were calculated for both of the depending variables (rMSSD and breathing rate). The first repeated-measures design was a 4 × 3 design (interventions Ujjayi, Paced Breathing, Kapalabhati, and Alternate Nostril Breathing × times t2, t3, and t4). See also Figure 1 and Figure 2. The second one was a 4 × 2 repeated-measures design (intervention x baseline measure t1, t2), testing whether the training itself effected rMSSD and breathing rate.

Type II sums of squares were used to test fixed effects (intervention, time, or baseline, and their interaction), and Satterthwaite’s method to approximate degrees of freedom. A random intercept was included for participant and a first-order autoregressive (ARI) covariance structure for repeated-measures residuals. Estimation via restricted maximum likelihood (REML) was assumed.

Six pairwise follow-up models were calculated to look further where the interaction between intervention and time or intervention and baseline became significant. Each was restricted to the two intervention levels being compared. Because of the six pairwise tests, a Bonferroni correction was applied, setting the significance threshold at α = 0.05/6 = 0.0083.

#### Data Exclusion

Table 1 details the number of valid observations at each measurement occasion, by technique. Missing data derived from three sources: (a) participant dropout (two participants withdrew after the first baseline measurement, as noted above); (b) isolated technical recording failures (e.g., ECG or respiration-belt signal loss during a single session); and (c) exclusion of rMSSD values falling outside the plausible physiological range of 19–75 ms [15]. The latter accounts for most of the additional N loss observed for Kapalabhati during practice (t3/t4: n = 24, compared to n = 31 at the immediately preceding baseline t2), consistent with the fact that Kapalabhati’s mild hyperventilation is expected to shift rMSSD toward the lower end of this range in some participants. All available data points were retained in the linear mixed models (i.e., participants were not excluded listwise), which is appropriate under the missing-at-random assumption of restricted maximum likelihood estimation.

## 3. Results

Results were presented for rMSSD and breathing frequency.

### 3.1. Heart-Rate Variability (rMSSD)

We examined whether there were significant changes in HRV over the time points t2, t3, and t4 of one measurement and also for differences between effects regarding the practiced breathing technique.

#### 3.1.1. Changes After Learning One Specific Breathing Technique

A significant main effect of the applied intervention was found (*F*(3, 101.82) = 4.12, *p* = 0.008 *), as well as a significant main effect of the time point of the measurement (*F*(2, 212.48) = 11.88, *p* < 0.001 *) and a significant interaction between intervention and time (*F*(6, 256.41) = 2.24, *p* = 0.040 *); see also Table 2.

The following six pairwise follow-up models compared changes in rMSSD over time between each pair of interventions (see Table 3). Results showed a significantly different course between Paced Breathing and Kapalabhati (*F*(2, 98.55) = 5.22, *p* = 0.007 *), and between Kapalabhati and Alternate Nostril Breathing (*F*(2, 98.52) = 10.23, *p* < 0.001 *) after Bonferroni correction (α = 0.0083). Other significance levels were over *p* ≥ 0.309. The general pattern of the data was similar within Ujjayi, Paced Breathing, and Alternate Nostril Breathing, with rMSSD increasing from baseline to the first part of the measurement during the intervention and decreasing slightly in comparison to the second part of the measurement. Kapalabhati showed a continuous, slight decrease over time. This shows its differences compared with the other techniques.

To see if any specific technique produces a significant change from baseline to practice, we computed direct within-technique contrasts for rMSSD and breathing frequency, comparing the baseline measurement (t2) with the first four minutes of practice (t3) and the second four minutes of practice (t4), and t3 with t4, separately for each of the four techniques. These contrasts were obtained as pairwise comparisons of estimated marginal means from linear mixed models fitted separately per technique (fixed effect: measurement occasion [t2/t3/t4]; repeated-measures covariance: first-order autoregressive, subject = participant). Mean differences, standard errors, degrees of freedom (Satterthwaite-approximated), and 95% confidence intervals are reported in Table 4 (rMSSD) and Table 5 (breathing frequency) below.

Because this analysis involves 4 techniques × 3 contrasts = 12 tests per dependent variable, we applied a Bonferroni-corrected significance threshold of α = 0.05/12 = 0.0042 across all 12 comparisons, consistent with the correction already applied to the six between-technique comparisons reported elsewhere in the manuscript (α = 0.0083).

The results show that the claim of a rapid rMSSD increase does not hold uniformly across all three slow-breathing techniques. The baseline-to-practice increase in rMSSD survived Bonferroni correction only for Paced Breathing (t2–t3) and for Alternate Nostril Breathing (t2–t3 and t2–t4); for Ujjayi, the increase was descriptively present but did not reach significance even without correction (*p* = 0.104), and Kapalabhati showed no significant change at any contrast.

#### 3.1.2. Changes in Pre-to-Post-Training Baseline

No significant effects emerged for intervention (*F*(3, 93.75) = 1.26, *p* = 0.291), Rest Period (*F*(1, 113.40) = 0.61, *p* = 0.438), or their interaction (*F*(3, 177.07) = 0.18, *p* = 0.908). All six pairwise follow-up comparisons were likewise non-significant (all *p* ≥ 0.062).

### 3.2. Breathing Frequency

We examined the same hypothesis of the rMSSD also regarding the breathing frequency, seeing whether there were significant changes in breathing frequency over the time points t2, t3, and t4 of one measurement, and also for differences between effects regarding the practiced breathing technique.

#### 3.2.1. Changes After Learning One Specific Breathing Technique

Regarding breathing frequency, we also found significant effects for intervention (*F*(3, 125.68) = 101.33, *p* < 0.001 *), time point, and the interaction between intervention and time (see Table 1).

Except for the differences between Ujjayi and Alternate Nostril Breathing, all follow-up pairwise comparisons were significant after Bonferroni correction (α = 0.0083): Ujjayi vs. Paced Breathing: *F*(2, 109.62) = 8.95, *p* < 0.001 *; Ujjayi vs. Kapalabhati: *F*(2, 116.10) = 38.78, *p* < 0.001 *; Ujjayi vs. Alternate Nostril Breathing: *F*(2, 106.73) = 0.06, *p* = 0.942; Paced Breathing vs. Kapalabhati: *F*(2, 108.80) = 70.44, *p* < 0.001 *; Paced Breathing vs. Alternate Nostril Breathing: *F*(2, 110.03) = 8.60, *p* < 0.001 *; and Kapalabhati vs. Alternate Nostril Breathing: *F*(2, 108.21) = 34.72, *p* < 0.001 * (see also Table 2).

Breathing frequency decreased from baseline to the first part of the measurement during practicing the breathing technique while practicing Ujjayi, Paced Breathing, and Alternate Nostril Breathing. Ujjayi and Alternate Nostril Breathing, the techniques that do not involve guiding the breathing from the outside, showed virtually identical courses. Kapalabhati showed a different pattern: the breathing frequency remained stable or slightly increased during the intervention phase.

#### 3.2.2. Changes in Pre-to-Post-Training Baseline

We found no significant effects for changes in breathing frequency while looking at differences between the baseline measure before training a breathing technique and after the training (main effect, intervention: *F*(3, 108.98) = 0.35, *p* = 0.786; main effect, rest period: *F*(1, 117.76) = 1.42, *p* = 0.235; and interaction, intervention × rest period: *F*(3, 188.10) = 2.23, *p* = 0.086). The six pairwise follow-up comparisons are reported for exploratory purposes, but they did not reach significance after Bonferroni correction (all *p* ≥ 0.063); see Table 2.

## 4. Discussion 

In this study, we found changes in heart-rate variability and breathing frequency through the use of breathing techniques. Although it is not surprising that breathing techniques affect those two variables, we examined whether there might be differences within subjects between different breathing techniques. As we expected, the data showed that one trained technique, Kapalabhati, accelerated the breathing frequency, while the three others prolonged it. Kapalabhati stood out from the other techniques, including its effect on rMSSD, which may indicate a different effect on the autonomic nervous system than the other techniques and, therefore, other possibilities for self-regulation in everyday life. This interpretation should be treated with caution, as no respiratory-volume and end-tidal CO_2_ data were collected.

Data on breathing frequency were nearly all significant when comparing different breathing techniques, except for the comparison between Ujjayi and Alternate Nostril Breathing. This is plausible because both techniques prolong breathing by narrowing the passage through which breath flows. In Ujjayi, this involves constricting the vocal cords, and in Alternate Nostril Breathing, air flows through one nostril rather than both. All other techniques differ regarding breathing frequency over time.

Even if most comparisons for breathing frequency over time reached significance, not all comparisons for changes in rMSSD over time were significant. Comparisons here showed the special effect of Kapalabhati, being different from Paced Breathing and the Alternate Nostril Breathing. The three techniques prolonging the breathing phases did not differ in their impact on the rMSSD over time, even though they differ in the development of breathing frequency over time.

We found no effect of any breathing technique between the baseline measures before and after the two weeks of training; this means the effect on the heart rate is achieved through direct practice and therefore influences breathing, at least after this short amount of practice time. The practicing effects of Paced Breathing and Alternate Nostril Breathing occur immediately, during the first half of practice. We found no benefit of longer practice time on rMSSD (comparing the first and second half of the practice). The results of Kapalabhati were different: rMSSD reduced slightly over time.

Paced Breathing and Alternate Nostril Breathing showed a stronger rapid rMSSD increase than Ujjayi when comparing the three slow breathing techniques. This suggests that not all slow breathing techniques lead to a rapid within-session increase in parasympathetic-associated HRV; in this study, this was most evident in techniques that explicitly guide the breathing cycle via audio guidance or nostril-switching. In contrast, breathing frequency decreased rapidly and consistently for all three slow techniques already within the first four minutes of practice, indicating that the respiratory change occurs immediately and uniformly even where the downstream cardiac-autonomic change does not.

The effect of breathing on HRV has been studied extensively [5,6,10,16]. Most studies examine the effects of one or two breathing techniques from a particular tradition. In the present study, four different (yoga) breathing techniques were examined, and indications for different effects of breathing techniques were found, particularly when categorized as techniques that slow down or speed up the breathing rhythm. The fact that all breathing techniques for slowing down the breathing rate increased HRV demonstrates how important these interventions are for research on self-regulation techniques for improving mental and physical well-being. Nevertheless, the question remains of how complex a breathing technique *must* be for everyday use, and to what extent breathing *must* be regulated downward to achieve effects.

The greatest change compared with baseline and in comparison to other interventions was achieved when using audio files in the Paced Breathing practice of intervention two. However, when interpreting the results, it should be taken into account that, apart from the experimental setup, the subjects were not confronted with direct stressors and were able to engage cognitively with their breathing without distraction. In challenging everyday situations, especially when thoughts are racing and causing stress, it can be helpful to give one’s mind a task to focus on. More complex breathing techniques (such as ANB) can be helpful here, as they provide a specific task to perform, interrupting and stopping catastrophizing and racing thoughts. Also, the audio files could take up this space and give the brain a cue to focus and bring attention back to self-awareness again. For future research, breathing techniques that engage the mind in different ways may be worth exploring. Also, it should be taken into account that, in certain groups, for example, among people with ADHD diagnosis, auditory stimuli like white and pink noise can help to improve task performance [35]. In this regard, the intensity of the effect may vary depending on the task instructions and the challenge that the brain must meet to implement them. Therefore, it may be interesting to examine differences in a person’s response to a specific breathing technique, taking into account neurological characteristics and personality traits.

Future studies could investigate how long the positive effects of the breathing techniques last by adding another baseline measure after the breathing practice.

In addition, the more experienced a person is in using conscious breath regulation, the faster the effect may occur. Studies have already shown that higher HRV improves stress regulation and leads to more pleasant feelings being reported [3,15,16].

As recent research increasingly links breathing and breathing patterns to cognitive, physiological, and emotional processes [36,37], a long-term study of breathing patterns in everyday life would help capture the full benefits that breathing training can have. To this end, subjects could go through their daily life wearing respiratory-belts that record their breathing movements while assessing events throughout the day through manual logging. Boyadzhieva and Kayhan (2021) [36] described how breathing modulates the assessment of stressors through various influencing factors: by consciously influencing an actually automatic process, the body can be tricked into a different state through the diversion of attention, bodily awareness, and the automatic influence on the autonomic nervous system and brain oscillations. By exploiting these connections, it may be possible to identify differences in how people with and without a mental health diagnosis deal with stress.

With a focus on breathing rate and breathing rhythm, other information could be discovered. Results from different reviews suggest that analyzing everyday breathing in connection with life events could provide insights into the mechanisms behind different ways of stimulus processing. Since breathing, in general, was found to influence the processing of emotional faces [36,37], this might also occur in emotional situations. It might be interesting to examine whether different breathing techniques have different impacts on the processing of emotional information.

Due to their influence on brain activity and different frequency bands, studies on the use of breathing techniques could be of great support in school contexts, for example [36]. A short breathing technique incorporated into lessons could help improve attention and help students process teaching material more effectively [19].

Even though the effect of breathing has become more of a focus in recent years, the question remains whether this effect has even more explanatory potential for intra-individual differences between people and their handling of challenging situations in everyday life and their regulatory abilities. As research in this area grows, breathing could play an even more important role in everyday life and in psychotherapy. This is particularly true given how easy and quick it is to use: just two weeks of training can already have a direct effect, with only 5 min of practice required. This can make breathing one of the most important tools for self-regulation.

### Limitations

One important limitation is the small sample size, which restricts the statistical Power of the analysis and increases the risk of a Type II error. Especially non-significant results should be interpreted with caution because they do not necessarily mean that there is no effect at all. Because of the small sample size and the limitations regarding training and measurements, no a priori power analysis was conducted. Besides the small sample size, there are limitations of this study that we need to mention. One is the relatively homogeneous sample. This makes it difficult to generalize the results. Cary-over effects cannot be fully excluded because of the design. Also, the first intervention, Ujjayi, was the basis of the training and always the first intervention in the training. Therefore, every following breathing technique is influenced by this part of the training, and results need to be interpreted in light of this issue. Nevertheless, because the three interventions were fully permuted after Ujjayi, carry-over effects can be expected to be less systematic than in a design with a fully fixed sequence of interventions for all participants. Another limitation might be the missing group without any respiratory focus in an intervention, done to exclude effects due only to breathing or sitting quietly. Because participants practiced the techniques, they were not blind to the experimental setup, and an expectancy effect is possible. Moreover, we did not examine changes in respiratory volume or end-tidal CO_2_ measurements, and for this reason, the results relating to the rMMSD should be interpreted with caution. It is also important to note that the experiment involved the use of a dry EEG cap. This was applied to the head with pressure, which caused some discomfort to the participants. Because of technical issues, these data cannot be presented. Despite this experimental setup, a relaxation effect was achieved during breathing for the participants. For this reason, it remains possible that, without this factor, the effects of the breathing techniques on physiology might have been even more distinct beneficially.

## Figures and Tables

**Figure 1:**
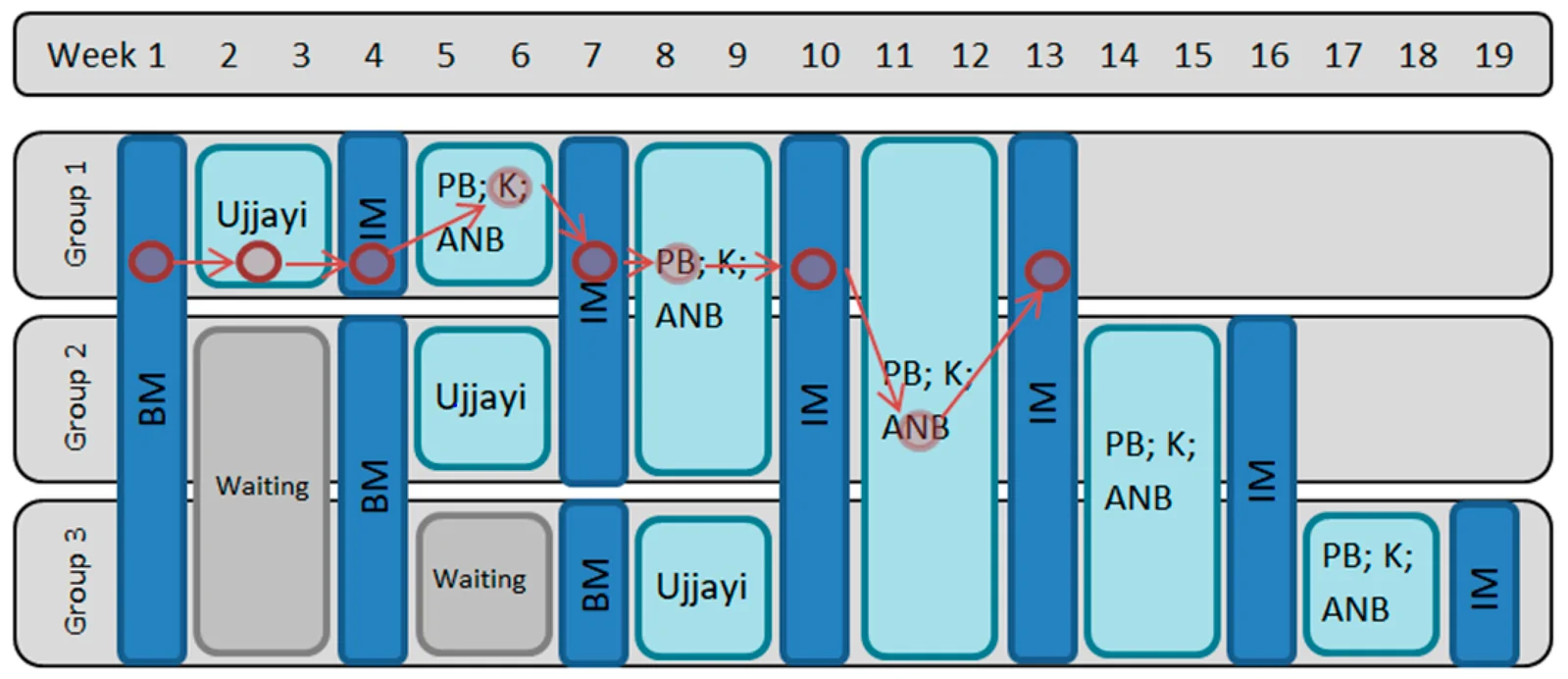
Training procedure for the three groups, including measurement time points. Baseline measurements (BMs) and intervention measurements (IMs) are marked (dark blue blocks). The first intervention, Ujjayi, is shown separately from the other three interventions, i.e., Paced Breathing (PB), Kapalabhati (K), and Alternate Nostril Breathing (ANB)(light-blue blocks). Points and arrows (red) show an example run-through by a test subject from group one with five measurement points and the sequence of interventions as follows: Ujjayi, Kapalabhati, Paced Breathing, and Alternate Nostril Breathing. The timeline and duration were illustrated using the week numbers shown above the diagram.

**Figure 2:**
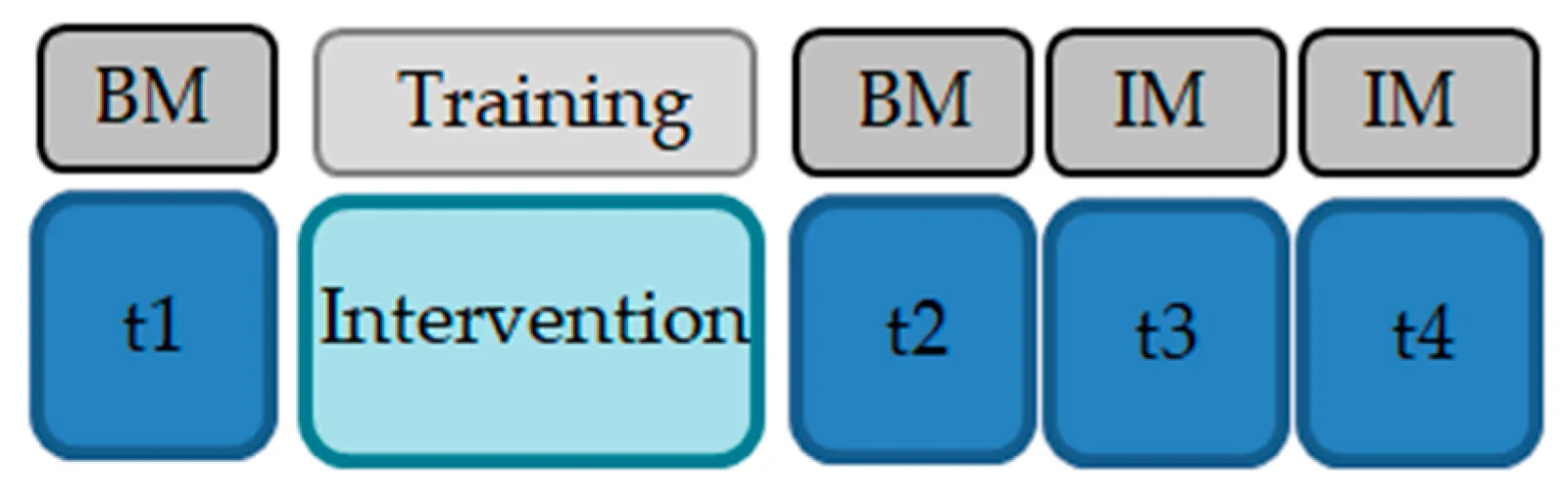
The available data structures from rMSSD and breathing frequency for the interventions: t1, baseline measure before training the specific intervention; t2, baseline measure after training the intervention; t3, first part of the measurement while practicing the intervention; and t4, second part of the measurement while practicing the intervention.

**Table 1:** Valid N and missing data by technique and measurement occasion. N = 36 participants enrolled. t1 = pre-training baseline (before the two-week training block for that technique); t2 = baseline immediately before practice; t3 = first four minutes of practice; t4 = second four minutes of practice. % miss. = percentage missing relative to N = 36.

Technique	t1 (Pre- Training) N	% Miss.	t2 (Baseline) N	% Miss.	t3 (First 4 Min) N	% Miss.	t4 (Second 4 Min) N	% Miss.
rMSSD (heart-rate variability)								
Ujjayi	30	17%	32	11%	32	11%	32	11%
Paced Breathing	30	17%	29	19%	30	17%	29	19%
Kapalabhati	30	17%	31	14%	24	33%	24	33%
Alternate Nostril Breathing	30	17%	29	19%	26	28%	26	28%
Breathing frequency								
Ujjayi	27	25%	34	6%	26	28%	26	28%
Paced Breathing	32	11%	32	11%	28	22%	28	22%
Kapalabhati	33	8%	33	8%	28	22%	28	22%
Alternate Nostril Breathing	32	11%	32	11%	25	31%	25	31%

Note. Listwise-complete cases across all 12 rMSSD variables (4 techniques × t2/t3/t4): *n* = 18. Listwise-complete cases across all 12 breathing-frequency variables (4 techniques × t2/t3/t4): *n* = 11.

**Table 2:** Fixed effects for the 4 × 3 (effect on intervention) and 4 × 2 (effect on baseline) linear mixed models of rMSSD and breathing frequency. Showing degrees of freedom (df), *F*-values, and *p*-values of the different effects, rMSSD = Root Mean Square of Successive Differences between normal heartbeats.

Model	Effect	Num. df	Den. df	F	*p*	η^2^p	Magnitude a
rMSSD 4 × 3	Intervention	3	101.82	4.12	0.008	0.108	Medium
rMSSD 4 × 3	Time	2	212.48	11.88	<0.001	0.101	Medium
rMSSD 4 × 3	Intervention × time	6	256.41	2.24	0.040	0.050	Small
rMSSD 4 × 2	Intervention	3	93.75	1.26	0.291	0.039	Small
rMSSD 4 × 2	Baseline	1	113.40	0.61	0.438	0.005	Very small
rMSSD 4 × 2	Intervention × baseline	3	177.07	0.18	0.908	0.003	Very small
Breathing frequency 4 × 3	Intervention	3	125.68	101.33	<0.001	0.707	Large
Breathing frequency 4 × 3	Time	2	213.69	209.35	<0.001	0.662	Large
Breathing frequency 4 × 3	Intervention × time	6	255.71	28.42	<0.001	0.400	Large
Breathing frequency 4 × 2	Intervention	3	108.98	0.35	0.786	0.010	Very small
Breathing frequency 4 × 2	Baseline	1	117.76	1.42	0.235	0.012	Small
Breathing frequency 4 × 2	Intervention × baseline	3	188.10	2.23	0.086	0.034	Small

Note. Degrees of freedom were approximated via Satterthwaite’s method. “Intervention” (4 × 3) models test the trend of the depending variable across baseline, the first part of the intervention, and the second part of the intervention. “Baseline” (4 × 2) models test pre-to-post-training change at rest. a: Magnitude classified using Cohen’s (1988) conventions for partial eta-squared: η^2^p ≈ 0.01 very small, 0.06 small, 0.14 medium/large boundary—here labeled very small (<0.01), small (0.01–0.05), medium (0.06–0.13), and large (≥0.14). η^2^p = (F × df1)/(F × df1 + df2).

**Table 3:** Pairwise follow-up comparisons for intervention × time/baseline interactions of rMSSD and breathing frequency. Showing degrees of freedom (df), *F*-values, and *p*-values of the different effects.

Comparison	Num. df	Den. df	F	*p*	η^2^p	Magnitude a
rMSSD, course during intervention						
Ujjayi vs. Paced Breathing	2	118.71	0.85	0.429	0.014	small
Ujjayi vs. Kapalabhati	2	109.34	1.15	0.321	0.021	small
Ujjayi vs. Alternate Nostril Breathing	2	104.36	1.19	0.309	0.022	small
Paced Breathing vs. Kapalabhati	2	98.55	5.22	0.007 *	0.096	medium
Paced Breathing vs. Alternate Nostril Breathing	2	106.68	0.09	0.911	0.002	very small
Kapalabhati vs. Alternate Nostril Breathing	2	98.52	10.23	<0.001 *	0.172	large
Breathing frequency, course during intervention						
Ujjayi vs. Paced Breathing	2	109.62	8.95	<0.001 *	0.140	large
Ujjayi vs. Kapalabhati	2	116.10	38.78	<0.001 *	0.400	large
Ujjayi vs. Alternate Nostril Breathing	2	106.73	0.06	0.942	0.001	very small
Paced Breathing vs. Kapalabhati	2	108.80	70.44	<0.001 *	0.564	large
Paced Breathing vs. Alternate Nostril Breathing	2	110.03	8.60	<0.001 *	0.135	medium
Kapalabhati vs. Alternate Nostril Breathing	2	108.21	34.72	<0.001 *	0.391	large
Breathing frequency, pre–post-baseline (exploratory)						
Ujjayi vs. Paced Breathing	1	79.18	4.71	0.033	0.056	small
Ujjayi vs. Kapalabhati	1	90.09	0.10	0.754	0.001	very small
Ujjayi vs. Alternate Nostril Breathing	1	84.14	1.52	0.220	0.018	small
Paced Breathing vs. Kapalabhati	1	92.95	3.53	0.063	0.037	small
Paced Breathing vs. Alternate Nostril Breathing	1	91.37	1.46	0.230	0.016	small
Kapalabhati vs. Alternate Nostril Breathing	1	92.68	0.99	0.323	0.011	small

Note. The omnibus intervention × baseline interaction for breathing frequency was not statistically significant (*p* = 0.086; see Table 1); these six comparisons are reported for exploratory purposes only, and none survived Bonferroni correction. Pairwise comparisons for rMSSD (4 × 2 model) are omitted because the corresponding omnibus interaction was not significant (smallest observed *p* = 0.554). a: Magnitude classified as in Table 1: very small (<0.01), small (0.01–0.05), medium (0.06–0.13), and large (≥0.14). The asterisk mark significant results.

**Table 4:** Within-technique contrasts of rMSSD (ms); t2 = baseline immediately before practice; t3 = first four minutes of practice; and t4 = second four minutes of practice. Mean difference = first level minus second level.

Technique	Contrast	Mean Diff.	SE	df	*p* (unadj.)	95% CI	Sig a
Ujjayi	t2–t3	−5.78	3.51	61.3	0.104	[−12.79, 1.23]	
Ujjayi	t2–t4	−1.16	4.43	82.4	0.794	[−9.98, 7.66]	
Ujjayi	t3–t4	4.62	3.51	61.3	0.193	[−2.39, 11.63]	
Paced Breathing	t2–t3	−11.56	2.29	55.4	<0.001	[−16.14, −6.97]	*
Paced Breathing	t2–t4	−6.96	3.03	68.5	0.025	[−13.00, −0.91]	
Paced Breathing	t3–t4	4.60	2.29	55.4	0.049	[0.01, 9.18]	
Kapalabhati	t2–t3	0.61	1.87	43.5	0.744	[−3.15, 4.37]	
Kapalabhati	t2–t4	2.98	2.56	49.1	0.250	[−2.17, 8.13]	
Kapalabhati	t3–t4	2.37	1.87	43.1	0.212	[−1.40, 6.13]	
Alternate Nostril Breathing	t2–t3	−12.90	2.28	47.2	<0.001	[−17.49, −8.30]	*
Alternate Nostril Breathing	t2–t4	−9.30	3.05	58.5	0.003	[−15.40, −3.20]	*
Alternate Nostril Breathing	t3–t4	3.59	2.29	46.5	0.123	[−1.01, 8.20]	

a: Significant at the Bonferroni-corrected threshold for 12 tests (α = 0.05/12 = 0.0042). The asterisk mark significant results.

**Table 5:** Within-technique contrasts of breathing frequency (breaths/minute); t2 = baseline immediately before practice; t3 = first four minutes of practice; and t4 = second four minutes of practice. Mean difference = first level minus second level.

Technique	Contrast	Mean Diff.	SE	df	*p* (unadj.)	95% CI	Sig a
Ujjayi	t2–t3	5.05	0.41	51.4	<0.001	[4.24, 5.87]	*
Ujjayi	t2–t4	5.00	0.53	66.9	<0.001	[3.95, 6.06]	*
Ujjayi	t3–t4	−0.05	0.41	49.0	0.907	[−0.87, 0.77]	
Paced Breathing	t2–t3	7.52	0.51	41.4	<0.001	[6.49, 8.56]	*
Paced Breathing	t2–t4	7.90	0.60	77.8	<0.001	[6.70, 9.10]	*
Paced Breathing	t3–t4	0.38	0.52	37.9	0.473	[−0.68, 1.44]	
Kapalabhati	t2–t3	−0.15	0.41	51.8	0.713	[−0.97, 0.67]	
Kapalabhati	t2–t4	−0.57	0.52	71.7	0.278	[−1.60, 0.47]	
Kapalabhati	t3–t4	−0.42	0.41	49.6	0.313	[−1.24, 0.41]	
Alternate Nostril Breathing	t2–t3	5.04	0.52	49.8	<0.001	[3.98, 6.09]	*
Alternate Nostril Breathing	t2–t4	4.93	0.62	74.2	<0.001	[3.69, 6.17]	*
Alternate Nostril Breathing	t3–t4	−0.10	0.54	44.7	0.847	[−1.18, 0.98]	

a: Significant at the Bonferroni-corrected threshold for 12 tests (α = 0.05/12 = 0.0042). The asterisk mark significant results.

## Data Availability

The original contributions presented in this study are included in the article/Supplementary Material. Further inquiries can be directed to the corresponding author.

## Supplementary Materials

The supporting information for participants can be downloaded at https://www.ulrichott.com/buecher/gesund-durch-atmen/ (accessed on 10 August 2026). On this page, you will find training videos to help you work through the training programme and practise the breathing techniques.
